# Differences in the efficacies of commonly used asthma questionnaires for predicting asthma-related outcomes among elderly and non-elderly patients in Japan

**DOI:** 10.1016/j.waojou.2025.101131

**Published:** 2025-12-10

**Authors:** Kazufumi Takada, Maho Suzukawa, Hiroyuki Tashimo, Nobuharu Ohshima, Masaki Ishii, Ken Ohta, the NHOM-Asthma study group

**Affiliations:** a Clinical Research Center, National Hospital Organization Tokyo National Hospital, 3-1-1 Takeoka, Kiyose-City, Tokyo 204-8585, Japan; b Department of Geriatric Medicine, Graduate School of Medicine, University of Tokyo, 7-3-1 Hongo, Bunkyo-Ku, Tokyo 113-8655, Japan; c Asthma, Allergy and Rheumatology Center, National Hospital Organization Tokyo National Hospital, 3-1-1 Takeoka, Kiyose-City, Tokyo 204-8585, Japan; d Center for Pulmonary Diseases, National Hospital Organization Tokyo National Hospital, 3-1-1 Takeoka, Kiyose-City, Tokyo 204-8585, Japan; e Japan Anti-Tuberculosis Association, JATA Fukujuji Hospital, 3-1-24 Matsuyama, Kiyose-City, Tokyo 204-8522, Japan

**Keywords:** Aged, Asthma, Elderly, Questionnaires, Respiratory function tests

## Abstract

**Background:**

Elderly asthma (EA) and non-elderly asthma (NEA) have been shown to differ greatly. The aim of this study was therefore to identify any differences in the abilities of commonly used asthma questionnaires to predict asthma outcomes in patients with EA and those with NEA, and to further determine the questions that are important for outcome prediction.

**Methods:**

This study analyzed data from the NHOM-Asthma study, a nationwide asthma registry in Japan which included 1014 patients with EA (aged ≥65 years) and 842 with NEA (aged <65 years). The correlations between total scores or individual items of the Asthma Control Questionnaire 6 (ACQ6), Asthma Quality of Life Questionnaire (AQLQ), and Asthma Starts with Knowledge 20, and asthma-related outcomes (unscheduled hospital visits, acute exacerbations, admissions within 1 year, and pulmonary function test results) were analyzed.

**Results:**

Worse baseline ACQ6 and AQLQ scores were significantly correlated with worse asthma-related outcomes in patients with NEA. In contrast, in patients with EA, these scores and the individual question items showed only a weak or no correlation with asthma-related outcomes. Instead, specific questions related to wheezing, nocturnal symptoms, nasal symptoms, and activity limitations were found to be closely associated with asthma-related outcomes in patients with EA. Moreover, the oscillometry data may better reflect the severity of asthma symptoms and activity limitations, as assessed by questionnaires, than spirometry data, including the forced expiratory volume in 1 sec, in patients with EA.

**Conclusions:**

Commonly used asthma questionnaires may not be suitable for predicting outcomes in patients with EA. However, specific questions addressing factors such as wheezing, nocturnal symptoms, nasal issues, and activity limitations may serve as reliable indicators of asthma-related outcomes in these patients.

## Introduction

Asthma is a common respiratory disease affecting up to 262 million people of all ages worldwide,^1^ though some reports suggest that this number could exceed 350 million.^2^ As a population ages, so do patients with asthma; this is especially relevant in Japan, which has the longest life expectancy.^3^ We and others have previously shown that elderly asthma (EA), in patients aged ≥65 years, and non-elderly asthma (NEA), in patients aged <65 years, differ greatly in terms of pathogenesis, symptoms, comorbidities, response to treatment, and mortality.^4^, ^5^, ^6^, ^7^, ^8^, ^9^ A variety of age-related factors affect the presentation of EA. These include anatomical and pathological changes within the lung, changes in the immune system, comorbidities, cognitive decline, weakening of skeletal muscles, and psychological effects. In addition, polypharmacy is more likely to be an issue in older patients, and can lead to poor medication adherence and an increased risk of drug-related adverse effects.^10^

Patient-reported questionnaires (PRQs) help to determine the level of disease control in individuals, and various asthma questionnaires are currently widely used.^11^, ^12^, ^13^, ^14^, ^15^ Questionnaires such as the Asthma Control Test and Asthma Control Questionnaire 6 (ACQ6) have been described by the Global Initiative for Asthma (GINA) as important tools for the evaluation of symptom control in asthma.^16^ However, most questionnaires are not age specific, and therefore may not be suitable for patients with EA.

Asthma PRQs comprise questions on symptom control, quality of life, and adherence to medication. We hypothesized that the question items that predict asthma-related outcomes may differ between EA and NEA. This study therefore aimed to identify question items that correlate with asthma-related outcomes in EA and NEA, which may aid in the development of a specific questionnaire for EA.

## Methods

### Study design

This study utilized data from NHOM-Asthma, a multicenter observational cohort study of asthma in Japan,^17^ the protocol and major findings of which have been previously published (registered in UMIN-CTR; UMIN000027776). The present study was approved by the Ethics Committee of the National Hospital Organization Tokyo National Hospital (Approval No. 220079). Written informed consent was obtained from all participants in NHOM-Asthma, and patient anonymity was preserved using methods approved by the Ethics Committee.

### Patients, data collection, and PRQs

Patients aged ≥18 years diagnosed and treated for asthma for more than 1 year were recruited from 27 national hospitals across Japan. Patients aged ≥65 years were defined as having EA and those aged <65 years were defined as having NEA. Clinical data was collected from medical charts, and the patients responded to the following PRQs: ACQ 6,^12^ a simple assessment of asthma symptom control; Asthma Quality of Life Questionnaire (AQLQ),^11^ which includes items assessing quality of life; Adherence Starts with Knowledge 20 (ASK-20),^18^ which assesses barriers to adherence; and Self-assessment of Allergic Rhinitis and Asthma (SACRA),^19^ a questionnaire on asthma and allergic rhinitis. These questionnaires (in Japanese) were distributed to outpatients at participating hospitals, filled out by patients at home, and submitted by mail. The only inclusion criterion for this secondary analysis was that patients had completed the PRQs at the time of enrollment.

Pulmonary function tests were also performed at enrollment. For patients with severe asthma (GINA step 4 or 5), the number of unscheduled hospital visits, acute exacerbations that required systemic corticosteroids, and admissions due to asthma were recorded for 1 year from the date of enrollment under the treatment based on the standard practices of their physicians.

### Statistical analysis

In this study, asthma-related outcomes were assessed using pulmonary function test results and the numbers of unscheduled hospital visits, acute exacerbations, and admissions during the one-year follow-up, as indicators of asthma remission.^20^ Univariate analysis was performed using linear regression models, with each question item used as an explanatory variable. The following asthma-related outcomes were treated as objective variables: percent predicted forced expiratory volume in 1 s (%FEV1), respiratory resistance (Rrs) at 5 Hz − Rrs at 20 Hz (R5−R20), respiratory reactance (Xrs) at 5 Hz (X5), low-frequency reactance area (ALX), resonant frequency (Fres), unscheduled hospital visits, acute exacerbations, admissions, and visual analogue scale (VAS) scores. All variables with a p-value ≤0.1 in univariate analysis were subsequently included in the multivariate analysis. To compare 2 groups, Fisher's exact test was used for categorical data and Wilcoxon's rank sum test was used for continuous data including one-year follow-up data. Spearman's correlation coefficients were calculated to examine the relationship between questionnaire scores and asthma-related outcomes. All statistical analyses were performed using R software version 3.6.2 (R Foundation for Statistical Computing, Vienna, Austria) or JMP Pro software version 16 (SAS Institute Japan, Tokyo, Japan).

## Results

### Baseline demographics and characteristics

Of the NHOM-Asthma study participants, 1856 completed the questionnaires at the time of enrollment and were included in the present study. Table 1 presents the baseline demographics and characteristics of the participants. Of the 1856 patients, 1014 had EA and 842 had NEA. Patients with EA were more likely to be male, have a history of smoking, and have a longer duration of asthma than those with NEA. Analysis of spirometry data showed that patients with EA had a lower FEV1, forced vital capacity (FVC), FEV1/FVC, and %FEV1 than those of patients with NEA. Oscillometry data showed higher R5−R20, lower X5, higher ALX, and higher Fres in patients with EA than in those with NEA. In terms of PRQs, patients with NEA reported lower AQLQ environment scores than those of patients with EA, indicating stronger asthma symptoms in response to environmental stimuli. Patients with EA scored lower in ASK-20 than did those with NEA, suggesting better adherence to treatment.

**Table 1:** Baseline patient characteristics.

Variables	EA (n = 1014)	NEA (n = 842)	P-value
Age at enrollment (years)	73 (69–78)	51 (42–58)	<0.001
Sex female, %	57.1	65.7	<0.001
BMI (kg/m^2^)	23.4 (21.2–25.8)	23.2 (20.7–26.5)	0.831
Age at asthma onset (years)	60 (45–66)	35 (18–47)	<0.001
Asthma duration (years)	13 (5–27)	10 (4–20)	<0.001
Smoking (pack-years)	0 (0–20)	0 (0–7.7)	<0.001
Respiratory function test			
FEV1 (mL)	1700 (1350–2110)	2520 (2070–2957.5)	<0.001
FVC (mL)	2510 (2060–3110)	3200 (2730–3827.5)	<0.001
FEV1/FVC (%)	69 (59–76)	79 (71–84)	<0.001
%FEV1 (%)^a^	92 (74–109)	97 (85–108)	<0.001
R5-R20 (cmH_2_O/L/s)	0.92 (0.56–1.4)	0.68 (0.3–1.16)	<0.001
X5 (cmH_2_O/L/s)	−0.75 (−1.65–−0.24)	−0.29 (−0.68–0.04)	<0.001
ALX (cmH_2_O/L)	3.44 (0.79–10.4)	1.01 (0.2–2.95)	<0.001
Fres (Hz)	10.48 (6.71–14.66)	6.83 (4.89–10.16)	<0.001
Questionnaire			
ACQ6 score	3 (1–7)	3 (0–7)	0.291
AQLQ activities	5.9 (5.2–6.5)	5.9 (5.3–6.6)	0.134
AQLQ symptoms	6.1 (5.5–6.8)	6.3 (5.5–6.8)	0.666
AQLQ emotions	6 (5.4–6.6)	6 (5.4–6.6)	0.745
AQLQ environment	6.3 (5.5–7)	6.3 (5.3–6.8)	0.010
AQLQ overall	6.1 (5.4–6.7)	6 (5.4–6.7)	0.365
ASK-20 score	34 (28–41)	37 (32–44)	<0.001
ASK-20 number of barriers	2 (1–4)	3 (1–5)	<0.001

Continuous data are expressed as median (interquartile range).

Abbreviations: ACQ6, Asthma Control Questionnaire 6; ALX, low-frequency reactance area; AQLQ, Asthma Quality of Life Questionnaire; ASK-20, Adherence Starts with Knowledge 20; BMI, body mass index; EA, elderly asthma; FEV1, forced expiratory volume in 1 s; Fres, resonant frequency; FVC, forced vital capacity; NEA, non-elderly asthma; R5−R20, respiratory resistance at 5 Hz − respiratory resistance at 20 Hz; X5, respiratory reactance at 5 Hz.

a %FEV1 was calculated by dividing FEV1 by predicted FEV1

The one-year follow-up data and characteristics of patients with severe asthma are shown in Table 2. Patients with severe EA reported significantly greater activity limitations than those of patients with severe NEA, whereas the opposite trend was observed in response to environmental stimuli. Patients with NEA had more unscheduled visits to hospital than those with EA; however, the numbers of acute exacerbations and admissions were comparable between the 2 groups.

**Table 2:** Characteristics of patients with severe asthma and asthma control during the one-year follow-up period.

	EA (n = 382)	NEA (n = 290)	P-value
Characteristics			
Age at enrollment (years)	74 (69–79)	52 (43–58)	<0.001
Sex female, %	56.5	64.1	0.048
Asthma duration (years)	16 (8–29)	11 (5–23)	<0.001
Smoking (pack-years)	0 (0–480)	0 (0–160)	0.002
Respiratory function test			
FEV1/FVC (%)	69 (58–76)	78 (68–84)	<0.001
%FEV1 (%)^a^	88 (71–107)	96 (79–108)	<0.001
R5-R20 (cmH_2_O/L/s)	1.00 (0.62–1.46)	0.78 (0.40–1.19)	<0.001
X5 (cmH_2_O/L/s)	−0.82 (−1.90–−0.21)	−0.19 (−0.72–0.18)	<0.001
ALX (cmH_2_O/L)	3.68 (0.67–12.90)	0.78 (0.08–3.20)	<0.001
Fres (Hz)	11.13 (6.50–16.42)	6.50 (4.45–10.33)	<0.001
Questionnaire			
ACQ6 score	5 (1–8)	5 (1–8)	0.784
AQLQ activities	5.5 (4.6–6.4)	5.7 (4.9–6.4)	0.047
AQLQ symptoms	5.9 (5.3–6.6)	5.8 (5.1–6.5)	0.535
AQLQ emotions	5.8 (5.0–6.6)	5.7 (5.0–6.4)	0.241
AQLQ environment	6.0 (5.5–6.8)	5.8 (4.8–6.7)	0.019
AQLQ overall	5.8 (5.1–6.4)	5.8 (5.1–6.4)	0.571
ASK-20 score	34 (29–41)	38 (32–46)	<0.001
ASK-20 number of barriers	2 (1–4)	3 (1–5)	<0.001
No. of unscheduled visits (%)			0.023
0	84.3	77.6	
1	9.4	11.4	
2	2.4	5.2	
3	1.6	2.8	
4	1.1	1.0	
5-9	1.0	2.1	
≥10	0.3	0.0	
No. of acute exacerbations (%)			0.108
0	70.7	65.5	
1	16.0	16.6	
2	5.0	5.9	
3	3.4	2.8	
4	0.5	2.4	
5-9	1.8	4.8	
≥10	2.6	2.1	
No. of admissions (%)			0.277
0	93.2	95.2	
1	4.7	3.8	
2	1.6	0.7	
3	0.3	0.0	
4	0.0	0.0	
≥5	0.3	0.3	

Continuous data are expressed as median (interquartile range).

Abbreviations: ACQ6, Asthma Control Questionnaire 6; ALX, low-frequency reactance area; AQLQ, Asthma Quality of Life Questionnaire; ASK-20, Adherence Starts with Knowledge 20; EA, elderly asthma; FEV1, forced expiratory volume in 1 s; Fres, resonant frequency; FVC, forced vital capacity; NEA, non-elderly asthma; R5−R20, respiratory resistance at 5 Hz − respiratory resistance at 20 Hz; X5, respiratory reactance at 5 Hz.

a %FEV1 was calculated by dividing FEV1 by predicted FEV1

### Correlations between PRQ scores and asthma-related outcomes

The correlations between frequently-used asthma PRQ scores and asthma-related outcomes are shown in Table 3. In patients with NEA, higher ACQ6 and lower AQLQ scores, which indicate worse asthma symptoms and asthma-attributed quality of life, were significantly correlated with the numbers of unscheduled hospital visits, acute exacerbations, admissions, and worse pulmonary function. However, in patients with EA, ACQ6 and AQLQ scores showed no or only weak correlation with asthma-related outcomes. ASK-20 scores were rarely correlated with asthma-related outcomes in patients with either EA or NEA.

**Table 3:** Correlations between individual PRQ scores and asthma-related outcomes.

ACQ6 score	EA		NEA	
	rho^a^	P-value	rho^a^	P-value
Unscheduled visits	0.091	0.086	**0.204**	<0.001
Acute exacerbations	0.187	<0.001	**0.301**	<0.001
Admissions	0.111	0.036	0.183	<0.001
%FEV1^b^	−0.170	<0.001	**−0.209**	<0.001
R5-R20	0.098	0.054	0.120	0.016
X5	−0.035	0.497	−0.117	0.019
ALX	0.039	0.443	0.117	0.019
Fres	0.036	0.480	0.120	0.016


AQLQ score	EA		NEA	
	rho^a^	P-value	rho^a^	P-value
Unscheduled visits	−0.104	0.086	**−0.256**	<0.001
Acute exacerbations	**−0.212**	<0.001	**−0.314**	<0.001
Admissions	−0.107	0.077	**−0.202**	0.001
%FEV1^b^	0.080	0.043	0.120	0.002
R5-R20	−0.058	0.329	−0.156	0.003
X5	−0.003	0.966	0.086	0.099
ALX	−0.003	0.964	−0.097	0.063
Fres	0.013	0.824	−0.083	0.112


ASK-20 score	EA		NEA	
	rho^a^	P-value	rho^a^	P-value
Unscheduled visits	0.138	0.013	−0.029	0.634
Acute exacerbations	0.091	0.104	−0.047	0.445
Admissions	0.078	0.163	0.012	0.843
%FEV1^b^	−0.042	0.258	−0.009	0.825
R5-R20	−0.046	0.385	0.097	0.060
X5	0.101	0.056	0.082	0.112
ALX	−0.101	0.055	−0.108	0.035
Fres	0.105	0.047	−0.118	0.021

Abbreviations: ACQ6, Asthma Control Questionnaire 6; ALX, low-frequency reactance area; AQLQ, Asthma Quality of Life Questionnaire; ASK-20, Adherence Starts with Knowledge 20; EA, elderly asthma; FEV1, forced expiratory volume in 1 s; Fres, resonant frequency; NEA, non-elderly asthma; PRQs, patient-reported questionnaires; R5−R20, respiratory resistance at 5 Hz − respiratory resistance at 20 Hz; X5, respiratory reactance at 5 Hz.

a Spearman's correlation coefficients are expressed as rho.

b %FEV1 was calculated by dividing FEV1 by predicted FEV1

### Association of asthma symptom question items with asthma-related outcomes

Next, the impact of each asthma PRQ question item on asthma-related outcomes was analyzed, with the results detailed in Supplemental Table 1–6. Table 4 presents the asthma symptom question items that were associated with asthma-related outcomes. In patients with EA, some question items were positively correlated with asthma-related outcomes, but unexpectedly, some were negatively correlated. For example, time of wheezes (ACQ6, No. 5), chest tightness (AQLQ, No. 6), and frequency of waking up at night due to asthma (AQLQ, No. 24) were significantly associated with an increased number of acute exacerbations and unscheduled hospital visits. Also, rhinitis symptoms, as determined by the SACRA questionnaire, were significantly associated with higher R5−R20, lower X5, and higher ALX results. In contrast, severity of shortness of breath due to asthma (ACQ6, No. 4) and frequency of chest heaviness (AQLQ, No. 14) were associated with fewer acute exacerbations and unscheduled hospital visits, respectively. In patients with NEA, severity of shortness of breath due to asthma (ACQ6, No. 4) and wheezes (ACQ6, No. 5, and AQLQ, No. 10) were significantly associated with decreased lung function.

**Table 4:** Asthma symptom question items associated with asthma-related outcomes.

Questionnaire	Question No.	Question item outline	Results (EA)	Results (NEA)
Wheeze				
ACQ-6	5	Time of wheezes (in last week)	The longer the wheeze, the more frequent the acute exacerbation	The longer the wheeze, the lower %FEV1^a^
AQLQ	10	Frequency of wheezes in the chest (in last 2 weeks)		The more frequent the wheeze, the higher Fres, higher R5-R20, lower X5, and higher ALX
Chest tightness				
AQLQ	6	Severity of discomfort or distress as a result of chest tightness (in last 2 weeks)	The greater the discomfort or distress, the more unscheduled visits	
AQLQ	14	Frequency of chest heaviness (in last 2 weeks)	The more frequent the chest heaviness, the less unscheduled visits	
Shortness of breath				
ACQ-6	4	Severity of shortness of breath due to asthma (in last week)	The more severe the shortness of breath, the less frequent the acute exacerbation	The more severe the shortness of breath, the lower %FEV1^a^
AQLQ	8	Frequency of shortness of breath due to asthma (in last 2 weeks)	The more frequent the shortness of breath, the more acute exacerbations, but fewer unscheduled visits	
Nocturnal symptoms				
AQLQ	24	Frequency of waking up at night due to asthma (in last 2 weeks)	The more frequent the waking up at night, the more unscheduled visits	
AQLQ	29	Frequency of asthma interfered with good night's sleep (in last 2 weeks)	The more frequent of the interferences, the more admissions, but higher %FEV1^a^	
Rhinitis symptom (SACRA)				
SACRA		Watery runny nose for more than 1 h almost every day		Patients with watery runny nose for more than 1 h almost every day have higher %FEV1^a^
SACRA		Stuffy nose for more than 1 h almost every day		Patients with stuffy nose for more than 1 h almost every day have higher Fres and higher ALX
SACRA		Itchy nose more than 1 h almost every day	Patients with itchy nose for more than 1 h almost every day have higher VAS, but higher %FEV1^a^	
SACRA		Rhinitis symptoms for more than 4 days per week		Patients with rhinitis symptoms for more than 4 days per week have higher VAS
SACRA		Degree of troublesome of rhinitis symptoms	The more troublesome the rhinitis symptoms, the higher R5-R20, lower X5, and higher ALX	

Abbreviations: ACQ6, Asthma Control Questionnaire 6; ALX, low-frequency reactance area; AQLQ, Asthma Quality of Life Questionnaire; EA, elderly asthma; FEV1, forced expiratory volume in 1 s; Fres, resonant frequency; NEA, non-elderly asthma; R5−R20, respiratory resistance at 5 Hz − respiratory resistance at 20 Hz; SACRA, Self-assessment of Allergic Rhinitis and Asthma; VAS, visual analogue scale; X5, respiratory reactance at 5 Hz.

a %FEV1 was calculated by dividing FEV1 by predicted FEV1

### Association of activity limitation question items with asthma-related outcomes

As shown in Table 5, in patients with EA, activity limitation was significantly associated with higher Fres (AQLQ, No. 1) and an increased number of admissions (AQLQ, No. 32). In patients with NEA, feeling the need to avoid dust (AQLQ, No. 19) and breathing symptoms restricting activities in school or work (SACRA) were significantly associated with unscheduled hospital visits.

**Table 5:** Activity limitation question items associated with asthma-related outcomes.

Questionnaire	Question No.	Question item outline	Results (EA)	Results (NEA)
AQLQ	1	Severity of limitation in intense activities (in last 2 weeks)	The severer the limitation, the higher Fres	
AQLQ	11	Frequency of feeling to avoid cigarette smoke (in last 2 weeks)	The more frequent the feeling to avoid cigarette smoke, the more acute exacerbations, but lower Fres	
AQLQ	19	Frequency of feeling to avoid dust (in last 2 weeks)		The more frequent the feeling to avoid dust, the more unscheduled visits
AQLQ	32	Degree of limitation of all activities due to asthma (in last 2 weeks)	The greater the degree of activity limitation, the more admissions	
SACRA		Breathing symptoms restrict activities in school or work (in last week)		Patients with breathing symptoms in last week have more unscheduled visits

Abbreviations: AQLQ, Asthma Quality of Life Questionnaire; EA, elderly asthma; Fres, resonant frequency; NEA, non-elderly asthma; SACRA, Self-assessment of Allergic Rhinitis and Asthma

### Association of emotional symptom and environmental stimulus question items with asthma-related outcomes

As shown in Table 6, in patients with EA, the frequency of feeling afraid of not having asthma medication available (AQLQ, No. 21) was significantly associated with the number of asthma admissions. In patients with NEA, exposure to strong smells or perfume (AQLQ, No. 26) was significantly associated with the number of acute exacerbations. In contrast, feeling afraid of getting out of breath (AQLQ, No. 27) was inversely associated with unscheduled hospital visits in patients with NEA.

**Table 6:** Emotional symptom and environmental stimulus question items associated with asthma-related outcomes.

Questionnaire	Question No.	Question item outline	Results (EA)	Results (NEA)
AQLQ	21	Frequency of feeling afraid of not having asthma medication available (in last 2 weeks)	The more frequent of the feeling of fear of not having medication, the more admissions	
AQLQ	26	Frequency of asthma symptoms by exposed to strong smells or perfume (in last 2 weeks)		The more frequent the symptoms caused by strong smells or perfume, the more acute exacerbations
AQLQ	27	Frequency of feeling afraid of getting out of breath (in last 2 weeks)		The more frequent the feeling of fear of getting out of breath, the less unscheduled visits

Abbreviations: AQLQ, Asthma Quality of Life Questionnaire; EA, elderly asthma; NEA, non-elderly asthma

### Association of ASK-20 question items with asthma-related outcomes

ASK-20 question items relevant to asthma-related outcomes are shown in Table 7. In patients with EA, the feeling of sadness was associated with more acute exacerbations and unscheduled hospital visits (ASK-20, No. 6). Stopping medication due to cost was significantly related to more unscheduled hospital visits (ASK-20, No. 19). However, there were also several unexpected results. For example, not having medicine when needed (ASK-20, No. 20) was significantly associated with lower Fres, higher X5, and lower ALX results. Alcohol getting in the way of taking medicines was associated with fewer acute exacerbations and unscheduled hospital visits in patients with EA, but inversely associated with unscheduled hospital visits in patients with NEA (ASK-20, No. 3). In patients with NEA, several ASK-20 question items, including not having someone to talk to about medicines (ASK-20, No. 9), not making decisions with doctor/nurse (ASK-20, No. 11) were significantly associated with worse pulmonary function.

**Table 7:** ASK-20 question items associated with asthma-related outcomes.

Questionnaire	Question No.	Question item outline	Results (EA)	Results (NEA)
ASK-20	1	Forgetting to take medicines some of the time		Patients who forget to take their medicines have higher %FEV1^a^
ASK-20	3	Using of alcohol gets in the way of taking medicines	Patients who cannot take medicines due to alcohol have fewer acute exacerbations and fewer unscheduled visits	Patients who cannot take medicines due to alcohol have more unscheduled visits and higher VAS
ASK-20	4	Worrying about how medicine will affect sexual health		Patients with anxiety about effect of medicine on sexual health have fewer acute exacerbations
ASK-20	6	Feeling sad, down, or blue during the past month	Patients who felt sad have more acute exacerbations and more unscheduled visits	
ASK-20	7	Feeling confident that each medicine will help	Patients with no trust in medicine have lower Fres	
ASK-20	9	Having someone who patients can call with questions about medicines		Patients with someone to talk to about medicines have lower R5-R20
ASK-20	11	Making decisions with doctor/nurse		Patients who make decisions with doctor/nurse have higher %FEV1^a^
ASK-20	13	Feeling inconvenient to take medicines more than once a day	Patients who feel inconvenient taking medicines more than once a day have lower %FEV1^a^	Patients who feel inconvenient taking medicines more than once a day have lower Fres and higher X5
ASK-20	15	Feeling hard to swallow pills		Patients who feel hard to swallow pills have higher VAS
ASK-20	16	Taking medicine more or less often than prescribed so far		Patients who have taken medicine more or less often than prescribed have lower ALX
ASK-20	19	Skipped, stopped, not refilled, or taken less medicine because of the cost so far	Patients who have taken off medicine due to cost have more unscheduled visits	
ASK-20	20	Not had medicine when it was time to take it	Patients who did not have their medicines at the correct time have lower Fres, higher X5, and lower ALX	

Abbreviations: ALX, low-frequency reactance area; EA, elderly asthma; FEV1, forced expiratory volume in 1 s; Fres, resonant frequency; NEA, non-elderly asthma; R5−R20, respiratory resistance at 5 Hz − respiratory resistance at 20 Hz; VAS, visual analogue scale; X5, respiratory reactance at 5 Hz.

a %FEV1 was calculated by dividing FEV1 by predicted FEV

## Discussion

The present study, conducted in Japan, demonstrated that, unlike patients with NEA, those with EA did not exhibit clear associations between frequently used asthma PRQs and asthma-related outcomes. Furthermore, the question items in these PRQs which correlated with asthma-related outcomes differed between patients with EA and those with NEA. In patients with EA, wheezing, nocturnal symptoms, and rhinitis were identified as key clinical symptoms. Furthermore, asthma-induced activity limitation was found to be a critical factor in predicting asthma-related outcomes. In patients with EA, oscillometry may better reflect the severity of asthma symptoms and activity limitations, as assessed by PRQs, than spirometry. Education on asthma medication appeared to be important for improving asthma-related outcomes, particularly in patients with EA.

Correlations between ACQ6 and AQLQ and asthma-related outcomes were robust in patients with NEA but minimal in those with EA. These PRQs were developed mainly for non-elderly patients,^11^^,^^12^ and indeed patients with chronic obstructive pulmonary disease (COPD) or heart failure, often comorbid with EA, were excluded during the development of ACQ6.^12^ Therefore, these PRQs may not accurately reflect asthma-related outcomes, particularly in Japan, where the population is aging rapidly. ASK-20 scores, the usefulness of which have been proven in Japanese patients with asthma,^21^ were minimally associated with these outcomes in patients with EA and NEA. This is not unexpected because ASK-20 was developed as a tool to improve adherence, not predict outcomes.^18^ Although asthma is a heterogenous disease and several distinct phenotypes exist in EA as distinguished from NEA,^3^^,^^22^, ^23^, ^24^, ^25^ a PRQ specific for EA may be necessary to accurately predict outcomes.

The present study determined the importance of specific question items in patients with EA and those with NEA. Unexpectedly, characteristic asthma symptoms including shortness of breath and chest heaviness did not correlate with worse asthma-related outcomes in patients with EA. The reason for this is unknown, but may be due to difficulties distinguishing asthma symptoms from those of comorbidities such as COPD and heart disease.^26^ Nevertheless, time of wheezes, frequency of waking up at night, and interference with sleep all showed a clear correlation with asthma-related outcomes which may be useful in assessing symptom control in patients with EA. Rhinitis question items were also useful for patients with EA and those with NEA, consistent with abundant evidence of the association between asthma and rhinitis from epidemiologic, pathophysiological and clinical studies.^27^^,^^28^

Limitations in daily activities predicted higher Fres and number of admissions in patients with EA. As physical activity is reduced in severe asthma,^29^, ^30^, ^31^ and older patients with asthma frequently report fatigue as their main symptom,^26^ activity limitation question items may be more critical for the management of EA than asthma symptom question items. Since absence from school or work were associated with unscheduled hospital visits in patients with NEA only, these question items may be irrelevant for patients with EA who are typically retired.

Another important finding of this study was that oscillometry may be a more useful asthma-related outcome in EA than spirometry. Indeed, %FEV1 often showed inverse relationships with asthma symptoms as assessed by PRQs, especially in patients with EA. Indicators of spirometry, i.e., FEV1 and FVC, decline with age,^32^^,^^33^ and spirometry is effort-dependent, and it is thus affected by frailty, cognitive or functional impairment, and physical difficulties such as scoliosis, kyphosis, or emaciation.^34^^,^^35^ Oscillometry is easier to perform and effort independent and therefore may be more useful as an asthma-related outcome in patients with EA. In this study, patients with EA exhibited higher respiratory resistance and more pronounced obstructive airflow limitation than those with NEA. While ALX, X5, and Fres have been associated with age, this association appears to be weak.^36^ The longer duration of asthma and the greater smoking history of patients with EA may significantly affect the current results, as a previous study demonstrated greater resistance and abnormality in the small airways in older patients with asthma.^7^

In patients with NEA, asthma symptoms were significantly associated with decreased FEV1 and worse oscillometry results, indicating the importance of pulmonary function as an asthma-related outcome in patients with NEA.

Finally, emotional symptom question items and adherence to treatment were found to be important for the management of EA. Feeling afraid of not having asthma medication and taking off medicine due to cost were associated with worse outcomes in patients with EA, suggesting the crucial importance of patient education on treatment adherence. Unexpectedly, patients with EA who did not trust in medications and those who did not have medicines at the correct time had better asthma-related outcomes. The reason for this is unclear, but patients with well-controlled or non-severe asthma may more easily withdraw from medications.

Interestingly, avoidance of strong odor and dust were closely associated with acute exacerbation or unscheduled hospital visits in patients with NEA but not in those with EA. Smelling a strong odor affects airway physiology and impacts asthma exacerbations,^37^ but since olfactory impairment is associated with age,^38^ odor may have less of an impact in EA.

Feeling sadness, a symptom of depression, was associated with worse asthma-related outcomes in patients with EA, which is consistent with previous findings showing that depression is common in older patients and can lead to poor adherence to asthma treatment.^39^ Moreover, cognitive decline is an important factor in older adults, and patients with cognitive impairment often have poor adherence to asthma treatment.^40^ Therefore, further multifaceted studies including cognitive function evaluation are necessary.

This study has several limitations. First, only patients with severe asthma (GINA step 4 or 5) were followed up, and changes in asthma medication during the follow-up period were not considered, although these may have influenced outcomes. Another limitation is the selection bias; this study was conducted in national hospitals in Japan, excluding patients with EA treated in care facilities or at home. Older patients vary in their ability to perform daily activities, frailty, and cognitive function. Therefore, the stratified analysis of large numbers of patients with EA remains necessary.

In conclusion, the efficacy of commonly used asthma PRQs is limited in EA. Activity limitation is a critical factor in predicting asthma-related outcomes in patients with EA. Furthermore, among the symptom-related items, wheezing, nocturnal symptoms, and nasal symptoms were identified as important prognostic factors in EA, distinguishing it from NEA. The present data indicate the need to develop a specific questionnaire for EA; however, this requires the performance of further studies including comprehensive geriatric assessments.

## Abbreviations

ACQ6, Asthma Control Questionnaire 6; ALX, low-frequency reactance area; AQLQ, Asthma Quality of Life Questionnaire; ASK-20, Adherence Starts with Knowledge 20; COPD, chronic obstructive pulmonary disease; EA, elderly asthma; FEV1, forced expiratory volume in 1 s; Fres, resonant frequency; FVC, forced vital capacity; GINA, Global Initiative for Asthma; NEA, non-elderly asthma; PRQs, patient-reported questionnaires; R5−R20, respiratory resistance at 5 Hz − respiratory resistance at 20 Hz; Rrs, respiratory resistance; SACRA, Self-assessment of Allergic Rhinitis and Asthma; VAS, visual analogue scale; X5, respiratory reactance at 5 Hz; Xrs, respiratory reactance.

## Ethics statement

This study was approved by the Institutional Review Board of the National Hospital Organization Tokyo National Hospital (Approval No. 220079). The requirement for informed consent was waived because this study was only based on the data from the previous study.

## Authors’ contributions

KT and MS conceived the study, performed the investigation, and visualized and analyzed the data. HT, NO, MI, and KO contributed to data curation. KT and MS wrote the original draft of the manuscript; KT, MS, HT, NO, MI, and KO reviewed and edited this draft. HT, NO, MI, and KO supervised the project and MS and KO administered the project. All authors read and approved the final manuscript.

## Consent for publication

All authors have read and agreed to the published version of the manuscript.

## Data availability

Not available.

## Submission declaration

Our manuscript is original, has not been published before, is not currently being considered for publication elsewhere.

## Funding source

None.

## Declaration of competing interest

MS received grants from Astellas, AstraZeneca, GlaxoSmithKline, Kyorin, Kyowa Kirin, MSD, Sanofi, and Shionogi, honoraria for lectures from AstraZeneca, Novartis Pharma, and Sanofi, and participated in an Advisory Board for AstraZeneca.
