# Supplementary material for: Differences in the efficacies of commonly used asthma questionnaires for predicting asthma-related outcomes among elderly and non-elderly patients in Japan
Source: World Allergy Organ J. 2025 Dec 10;18(12):101131. doi: 10.1016/j.waojou.2025.101131 (PMC12753502; doi:10.1016/j.waojou.2025.101131)
Supplement: Multimedia component 1 [file mmc1.docx]

Supplemental Table 1. Multivariate analysis of the association between question items and asthma exacerbation

| Patients | Questionnaire | Question No. | Question item outline | Scoring | Regression coefficient | 95% CI | P-value |
| --- | --- | --- | --- | --- | --- | --- | --- |
| EA | ACQ6 | 4 | Shortness of breath (in last week) | 0: none -  6; a very great deal | -0.636 | -1.043, -0.229 | 0.002 |
|  | ACQ6 | 5 | Time of wheezes (in last week) | 0; not at all -  6; all the time | 0.524 | 0.145, 0.902 | 0.007 |
|  | AQLQ | 8 | Feeling short of breath due to asthma (in last 2 weeks) | 1; all of the time -  7; none of the time | -0.427 | -0.774, -0.08 | 0.016 |
|  | AQLQ | 11 | Feeling you had to avoid cigarette smoke (in last 2 weeks) | 1; all of the time -  7; none of the time | -0.145 | -0.28, -0.009 | 0.037 |
|  | ASK-20 | 3 | Use of alcohol gets in the way of taking medicines | 1; strongly disagree -  5; strongly agree | -0.586 | -1.146,  -0.026 | 0.040 |
|  | ASK-20 | 6 | Feeling sad, down, or blue (in last month) | 1; strongly disagree -  5; strongly agree | 0.299 | 0.055, 0.543 | 0.016 |
|  | SACRA |  | Use of rescue inhaler in a week (>3 times) | Yes | 1.065 | 0.044, 2.087 | 0.041 |
| NEA | AQLQ | 26 | Asthma symptoms by strong smells or perfume (in last 2 weeks) | 1; all of the time -  7; none of the time | -0.209 | -0.415, -0.003 | 0.047 |
|  | ASK-20 | 4 | Worry about how medicine will affect my sexual health | 1; strongly disagree -  5; strongly agree | -0.255 | -0.509,  -0.001 | 0.049 |
|  | SACRA |  | Asthma symptoms in a week (>3 times) | Yes | 1.527 | 0.668, 2.385 | 0.001 |

Abbreviations: ACQ6, Asthma Control Questionnaire 6; AQLQ, Asthma Quality of Life Questionnaire; ASK-20, Adherence Starts with Knowledge 20; CI, confidence interval; EA, elderly asthma; NEA, non-elderly asthma; SACRA, Self-assessment of Allergic Rhinitis and Asthma

Supplemental Table 2. Multivariate analysis of the association between question items and unscheduled hospital visits

| Patients | Questionnaire | Question No. | Question item outline | Scoring | Regression coefficient | 95% CI | P-value |
| --- | --- | --- | --- | --- | --- | --- | --- |
| EA | AQLQ | 6 | Feeling discomfort or distress as a result of chest tightness (in last 2 weeks) | 1; a very great deal - 7; no discomfort or distress | -0.204 | -0.397,  -0.011 | 0.038 |
|  | AQLQ | 8 | Feeling short of breath as a result of asthma (in last 2 weeks) | 1; all of the time -  7; none of the time | 0.221 | 0.049, 0.392 | 0.012 |
|  | AQLQ | 14 | Feeling chest heaviness (in last 2 weeks) | 1; all of the time -  7; none of the time | 0.263 | 0.053, 0.473 | 0.014 |
|  | AQLQ | 24 | Night awakening due to asthma (in last 2 weeks) | 1; all of the time -  7; none of the time | -0.311 | -0.514,  -0.109 | 0.003 |
|  | AQLQ | 30 | Feeling of fighting for air (in last 2 weeks) | 1; all of the time -  7; none of the time | 0.251 | 0.035, 0.468 | 0.023 |
|  | ASK-20 | 3 | Use of alcohol gets in the way of taking medicines | 1; strongly disagree -  5; strongly agree | -0.363 | -0.647,  -0.078 | 0.013 |
|  | ASK-20 | 6 | Feeling sad, down, or blue (in last month) | 1; strongly disagree -  5; strongly agree | 0.153 | 0.029, 0.276 | 0.015 |
|  | ASK-20 | 19 | Skipped, stopped, taken less medicine because of the cost | 1; never -  5; in the last week | 0.475 | 0.134, 0.815 | 0.006 |
|  | SACRA |  | Asthma symptoms in the last week (>3 times) | Yes | 0.927 | 0.301, 1.553 | 0.004 |
| NEA | AQLQ | 19 | Feeling you had to avoid dust (in last 2 weeks) | 1; all of the time -  7; none of the time | -0.115 | -0.2,  -0.031 | 0.008 |
|  | AQLQ | 27 | Feeling afraid of getting out of breath (in last 2 weeks) | 1; all of the time -  7; none of the time | 0.191 | 0.044, 0.339 | 0.011 |
|  | AQLQ | 30 | Feeling of fighting for air (in last 2 weeks) | 1; all of the time -  7; none of the time | 0.244 | 0.059, 0.43 | 0.010 |
|  | ASK-20 | 3 | Use of alcohol gets in the way of taking medicines | 1; strongly disagree -  5; strongly agree | 0.222 | 0.035,  0.408 | 0.020 |
|  | SACRA |  | Breathing symptoms restrict activities in school or work (in last week) | Yes | 0.758 | 0.071, 1.444 | 0.031 |
|  | SACRA |  | Use of rescue inhaler in the last week (1-2 times) | Yes | 0.506 | 0.064, 0.949 | 0.025 |
|  | SACRA |  | Use of rescue inhaler in the last week (>3 times) | Yes | 0.588 | 0.028, 1.148 | 0.040 |

Abbreviations: AQLQ, Asthma Quality of Life Questionnaire; ASK-20, Adherence Starts with Knowledge 20; CI, confidence interval; EA, elderly asthma; NEA, non-elderly asthma; SACRA, Self-assessment of Allergic Rhinitis and Asthma

Supplemental Table 3. Multivariate analysis of the association between question items and admission

| Patients | Questionnaire | Question No. | Question item outline | Scoring | Regression coefficient | 95% CI | P-value |
| --- | --- | --- | --- | --- | --- | --- | --- |
| EA | AQLQ | 20 | Wake up in the morning with asthma symptoms (in last 2 weeks) | 1; all of the time -  7; none of the time | 0.1 | 0.008, 0.193 | 0.034 |
|  | AQLQ | 21 | Feeling afraid of not having medication available (in last 2 weeks) | 1; all of the time -  7; none of the time | -0.079 | -0.141,  -0.017 | 0.013 |
|  | AQLQ | 29 | Interference with good night's sleep (in last 2 weeks) | 1; all of the time -  7; none of the time | -0.135 | -0.236,  -0.034 | 0.009 |
|  | AQLQ | 30 | Feeling of fighting for air (in last 2 weeks) | 1; all of the time -  7; none of the time | 0.159 | 0.034, 0.284 | 0.013 |
|  | AQLQ | 32 | Limited in all the activities due to asthma (in last 2 weeks) | 1; Totally limited, couldn’t do activity at all - 7; not at all limited | -0.119 | -0.226,  -0.012 | 0.030 |
| NEA | SACRA |  | Asthma symptoms in the last week (>3 times) | Yes | 0.255 | 0.062, 0.448 | 0.010 |

Abbreviations: AQLQ, Asthma Quality of Life Questionnaire; CI, confidence interval; EA, elderly asthma; NEA, non-elderly asthma; SACRA, Self-assessment of Allergic Rhinitis and Asthma

Supplemental Table 4. Multivariate analysis of the association between question items and asthma visual analog scale

| Patients | Questionnaire | Question No. | Question item outline | Scoring | Regression coefficient | 95% CI | P-value |
| --- | --- | --- | --- | --- | --- | --- | --- |
| EA | SACRA |  | Having itchy nose more than one hour almost every day | Yes | 5.754 | 1.119, 10.389 | 0.015 |
| NEA | ASK-20 | 3 | Use of alcohol gets in the way of taking medicines | 1; strongly disagree -  5; strongly agree | 3.431 | 0.701, 6.161 | 0.014 |
|  | ASK-20 | 15 | Feeling hard to swallow the pills | 1; strongly disagree -  5; strongly agree | 3.316 | 1.176, 5.456 | 0.002 |
|  | SACRA |  | Having symptoms of rhinitis more than 4 days per week | Yes | 7.255 | 3.059, 11.451 | 0.001 |

Abbreviations: ASK-20, Adherence Starts with Knowledge 20; CI, confidence interval; EA, elderly asthma; NEA, non-elderly asthma; SACRA, Self-assessment of Allergic Rhinitis and Asthma

Supplemental Table 5. Multivariate analysis of the association between question items and %FEV1

| Patients | Questionnaire | Question No. | Question item outline | Scoring | Regression coefficient | 95% CI | P-value |
| --- | --- | --- | --- | --- | --- | --- | --- |
| EA | AQLQ | 29 | Interference with good night's sleep (in last 2 weeks) | 1; all of the time -  7; none of the time | -6.482 | -12.052,  -0.912 | 0.023 |
|  | ASK-20 | 13 | Feeling inconvenient to take medicines more than once a day | 1; strongly disagree -  5; strongly agree | -3.537 | -6.586,  -0.488 | 0.023 |
|  | SACRA |  | Having itchy nose more than one hour almost every day | Yes | 14.682 | 3.84, 25.525 | 0.008 |
| NEA | ACQ6 | 4 | Shortness of breath (in last week) | 0: none -  6; a very great deal | -3.245 | -5.991,  -0.5 | 0.021 |
|  | ACQ6 | 5 | Time of wheezes (in last week) | 0; not at all -  6; all the time | -3.774 | -6.804,  -0.744 | 0.015 |
|  | ASK-20 | 1 | Forgetting to take medicines some of the time | 1; strongly disagree -  5; strongly agree | 2.721 | 0.836, 4.606 | 0.005 |
|  | ASK-20 | 11 | Working together with doctor/nurse to make decisions | 1; strongly agree -  5; strongly disagree | -5.719 | -8.937,  -2.501 | 0.001 |
|  | SACRA |  | Watery runny nose for more than one hour almost every day | Yes | 7.452 | 1.845, 13.059 | 0.010 |

Abbreviations: ACQ6, Asthma Control Questionnaire 6; AQLQ, Asthma Quality of Life Questionnaire; ASK-20, Adherence Starts with Knowledge 20; CI, confidence interval; EA, elderly asthma; NEA, non-elderly asthma; SACRA, Self-assessment of Allergic Rhinitis and Asthma

Supplemental Table 6. Multivariate analysis of the association between question items and Fres

| Patients | Questionnaire | Question No. | Question item outline | Scoring | Regression coefficient | 95% CI | P-value |
| --- | --- | --- | --- | --- | --- | --- | --- |
| EA | AQLQ | 1 | Limited in intense activity due to asthma (in last 2 weeks) | 1; Totally limited, couldn’t do activity at all - 7; not at all limited | -0.821 | -1.402,  -0.24 | 0.006 |
|  | AQLQ | 11 | Feeling you had to avoid cigarette smoke (in last 2 weeks) | 1; all of the time -  7; none of the time | 0.591 | 0.123, 1.06 | 0.014 |
|  | ASK-20 | 7 | Feeling confident that medicines help you | 1; strongly agree -  5; strongly disagree | -1.473 | -2.476,  -0.469 | 0.004 |
|  | ASK-20 | 20 | Not had medicine when it was time to take it | 1; never -  5; in the last week | -1.331 | -2.251,  -0.412 | 0.005 |
| NEA | AQLQ | 10 | Experiencing a wheeze in chest (in last 2 weeks) | 1; all of the time -  7; none of the time | -1.137 | -1.766,  -0.507 | 0.001 |
|  | AQLQ | 16 | Feeling the need to clear the throat (in last 2 weeks) | 1; all of the time -  7; none of the time | 0.889 | 0.396, 1.382 | 0.001 |
|  | ASK-20 | 13 | Feeling inconvenient to take medicines more than once a day | 1; strongly disagree -  5; strongly agree | -0.611 | -1.097,  -0.125 | 0.014 |
|  | SACRA |  | Stuffy nose for more than one hour almost every day | Yes | 1.845 | 0.44, 3.249 | 0.011 |

Abbreviations: AQLQ, Asthma Quality of Life Questionnaire; ASK-20, Adherence Starts with Knowledge 20; CI, confidence interval; EA, elderly asthma; NEA, non-elderly asthma; SACRA, Self-assessment of Allergic Rhinitis and Asthma
